# Overcoming the Blood–Brain Tumor Barrier with Docetaxel-Loaded Mesoporous Silica Nanoparticles for Treatment of Temozolomide-Resistant Glioblastoma

**DOI:** 10.1021/acsami.4c04289

**Published:** 2024-04-17

**Authors:** Tsung-I Hsu, Yi-Ping Chen, Rong-Lin Zhang, Zih-An Chen, Cheng-Hsun Wu, Wen-Chang Chang, Chung-Yuan Mou, Hardy Wai-Hong Chan, Si-Han Wu

**Affiliations:** † Ph.D. Program in Medical Neuroscience, College of Medical Science and Technology, Taipei Medical University and National Health Research Institutes, Taipei 110, Taiwan; ‡ International Master Program in Medical Neuroscience, College of Medical Science and Technology, Taipei Medical University, Taipei 110, Taiwan; § Graduate Institute of Nanomedicine and Medical Engineering, Taipei Medical University, Taipei 110, Taiwan; ∥ International Ph.D. Program in Biomedical Engineering, College of Biomedical Engineering, Taipei Medical University, Taipei 110, Taiwan; ⊥ Nano Targeting & Therapy Biopharma Inc., Taipei 110, Taiwan; # Graduate Institute of Medical Sciences, College of Medicine, Taipei Medical University, Taipei 110, Taiwan; ∇ Department of Chemistry, National Taiwan University, Taipei 106, Taiwan

**Keywords:** blood−brain tumor barrier, docetaxel, mesoporous silica nanoparticles, temozolomide resistant, glioblastoma

## Abstract

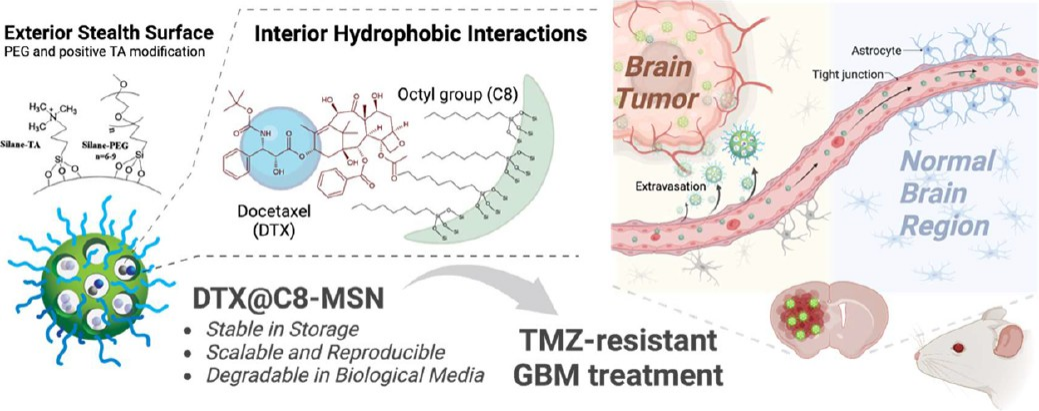

While temozolomide (TMZ) has been a cornerstone in the treatment of newly diagnosed glioblastoma (GBM), a significant challenge has been the emergence of resistance to TMZ, which compromises its clinical benefits. Additionally, the nonspecificity of TMZ can lead to detrimental side effects. Although TMZ is capable of penetrating the blood–brain barrier (BBB), our research addresses the need for targeted therapy to circumvent resistance mechanisms and reduce off-target effects. This study introduces the use of PEGylated mesoporous silica nanoparticles (MSN) with octyl group modifications (C8-MSN) as a nanocarrier system for the delivery of docetaxel (DTX), providing a novel approach for treating TMZ-resistant GBM. Our findings reveal that C8-MSN is biocompatible in vitro, and DTX@C8-MSN shows no hemolytic activity at therapeutic concentrations, maintaining efficacy against GBM cells. Crucially, in vivo imaging demonstrates preferential accumulation of C8-MSN within the tumor region, suggesting enhanced permeability across the blood–brain tumor barrier (BBTB). When administered to orthotopic glioma mouse models, DTX@C8-MSN notably prolongs survival by over 50%, significantly reduces tumor volume, and decreases side effects compared to free DTX, indicating a targeted and effective approach to treatment. The apoptotic pathways activated by DTX@C8-MSN, evidenced by the increased levels of cleaved caspase-3 and PARP, point to a potent therapeutic mechanism. Collectively, the results advocate DTX@C8-MSN as a promising candidate for targeted therapy in TMZ-resistant GBM, optimizing drug delivery and bioavailability to overcome current therapeutic limitations.

## 1 Introduction

Glioblastoma (GBM) remains highly lethal despite surgical resection, concurrent radiation, and chemotherapy, as well as adjuvant temozolomide (TMZ) therapies.^1,2^ The recurrence rate of GBM is over 90%. This stems from the fact that gliomas are characteristically diffuse with infiltrating edges, resistant to drugs, and nearly inaccessible to systemic therapies due to the blood–brain barrier (BBB).^3^ Biologics and more than 98% of small molecules do not cross BBB. In most cases, brain tumor expansion forms pathological vasculature (i.e., blood–brain tumor barrier, BBTB) with increased fenestrations compared to the BBB.^4,5^ However, this vascular structural change is insufficient to allow drug penetration; thus, BBTB continues to be a major obstacle to drug delivery.^5^ Moreover, the prompt removal of drugs through active transport can hinder drug penetration. Even though new potent agents are available, drug delivery to brain tumors is limited, most often leading to a poor prognosis of either primary or metastatic brain tumors.

TMZ has been a first-line chemotherapy drug for glioma treatment for over a decade; it was first approved by the FDA in 2005.^1^ When combined with radiation therapy and employed as adjuvant therapy, TMZ treatment is able to extend the survival period of tumor-excised GBM patients.^2^ Unfortunately, in patients with recurrent GBM, TMZ treatment resistance increases, with a five-year survival rate of 5.8% and an average survival period of less than 2 years.^6^ These poor outcomes underscore the urgent need for developing new therapeutic strategies for treating TMZ-resistant GBM.

Advances in nanomedicine including nanodrug delivery systems (NDDS) hold immense potential to revolutionize the delivery of therapeutics to tumors.^7,8^ To date, few NDDS can penetrate BBB/BBTB effectively and safely.^9,10^ Mesoporous silica nanoparticles (MSN) have great potential as drug delivery systems due to their unique physical/chemical properties, such as large pore volume, chemical/thermal stability, high loading capacity, adjustable surface properties, and excellent biocompatibility.^11−14^ Notably, it has been reported that surface-modified silica nanoparticles can penetrate BBB very well.^15−17^ Leveraging this knowledge, our study employs a carefully optimized surface modification strategy on MSNs, designed to enhance BBB penetration and tumor targeting via the enhanced permeability and retention (EPR) effect, an approach validated by our previous research to significantly improve drug delivery to brain tumors.^18^ In this report, we demonstrate the efficacy of an MSN carrier loaded with the FDA-approved cancer drug docetaxel (DTX), for the treatment of TMZ-resistant brain tumors in a mouse model.

Docetaxel is a semisynthetic analog of paclitaxel, an extract from a Pacific yew tree (*Taxus brevifolia*).^19,20^ DTX prevents physiological microtubule depolymerization and disassembly, leading to cell cycle arrest in the G2/M phase and cell death. A commercial DTX injection, taxotere, is formulated in nonionic surfactant polysorbate 80 (Tween 80) and 13% ethanol solution. The therapeutic benefits of combining Taxotere with other antineoplastic agents in the clinic have been verified and approved for several cancer indications, including breast, prostate, nonsmall cell lung, head and neck cancers, and gastric adenocarcinoma. Notably, however, brain cancer is not on the list despite studies that showed that DTX is effective against human U87MG-glioblastoma cancer cells in vitro.^21^ However, the poor intrinsic BBB/BBTB permeability and off-target cytotoxic effects of DTX have limited its utility in patients with recurrent malignant glioblastoma.^22,23^ Furthermore, taxotere has been associated with injection site reactions and systemic adverse reactions, such as hypersensitivity, nonallergic anaphylaxis, and rash.^24,25^ Tumor types that are less drug-accessible have greater DTX-associated systemic toxicity, which is dose-limiting and has led to failure in many clinical trials. Thus, an ideal NDDS for brain tumors such as GBM would need (i) good drug carrier ability for DTX, (ii) good BBTB penetration, and (iii) less systemic toxicity.

Recently, there have been efforts to utilize dendrimer formulations as the NDDS for combating brain diseases.^26^ In vivo anticancer activity in brain tumor-bearing rats revealed that DTX-loaded P80 conjugated poly(propyleneimine) (PPI) dendrimers significantly reduced tumor volume.^21^ Dendrimers owe their ability to penetrate the BBB/BBTB to their very small size (∼20 nm), which other nanoparticles such as liposomes or lipid nanoparticles cannot reach. Unfortunately, it has been reported that cationic dendrimers like poly(amido amine) (PAMAM), PPI, and poly l-lysine have demonstrated toxicity in a dose-dependent manner.^27^ Strategies, such as building charge-reversal and hierarchically structuring dendrimers, are being evaluated to determine the feasibility of dendrimers in medicine.^28^

Apart from dendrimers, Gregory et al. synthesized a synthetic protein nanoparticle (SPNP) consisting of polymerized human serum albumin (HSA), coloaded with the tumor cell-penetrating peptide iRGD and siRNA against a transcriptional activator (STAT3) associated with GBM progression.^29^ While the accumulation of iRGD-loaded SPNPs was 40% higher in the brains of tumor-bearing mice than in nontumor-bearing mice, the distribution of SPNPs within the brain was minimal compared to that in other organs. Using HSA as a nanocarrier would not be suitable for a toxic drug such as DTX because it would still lead to systemic toxicity. In contrast, to enhance BBB penetration, a study employed angiopep-2-docked polymersome NDDS for the brain delivery of volasertib, a Plk1 inhibitor, showcasing potent antitumor effects and increased survival in mice.^9^ Additionally, Wei et al. leveraged this polymersome strategy to introduce a CpG nanoimmunoadjuvant for noninvasive immunotherapy of malignant glioma, further exemplifying the potential of polymersomes in achieving targeted delivery across the BBB.^10^ Other delivery efforts such as a liposomal TMZ formulation across the BBTB to GBM were made in combination with ultrasound-mediated BBB opening^30^ or convection-enhanced delivery (CED).^31^ Although variable levels of successful ultrasound-mediated BBB disruption were achieved, no clear-cut advantages or long-term safety studies have been reported.^32^

We herein report a nanodrug delivery system built with MSN of hydrodynamic diameter below 45 nm with (1) an external functionalization of PEG and quaternary ammonium groups for good blood circulation and (2) an internal hydrophobic functionalization for good drug loading of DTX. Both the drug loading and release behavior in the physiological solution were investigated. Thanks to the confinement effect of the mesoporous channels, DTX exhibits significantly enhanced solubility when it is adsorbed molecularly and stabilized in an amorphous form rather than a crystalline form. In vivo two-photon imaging has shown that the small particle size and external surface functionalization facilitate effective penetration of BBTB. When combined with intraperitoneal administration of TMZ, intravenous injections of DTX@C8-MSN resulted in almost complete tumor shrinkage in U87 tumor mice that were resistant to TMZ. This treatment led to a notable rise of 51.9% in the life span percentage.

## 2 Materials and Methods

### 2.1 Materials

All materials were used without further purification. Triethoxy(octyl)silane (97%) and rhodamine B isothiocyanate (RITC) were purchased from Sigma-Aldrich (Milwaukee, WI). Cetyltrimethylammonium bromide (CTAB, 99+%), tetraethyl orthosilicate (98%), ammonium hydroxide (NH_4_OH, 28–30 wt % as NH_3_), hydrochloric acid (for analysis, fuming, 37% solution in water) were purchased from ACROS. 2-[Methoxy(polyethyleneoxy)6–9propyl]trimethoxysilane, tech-90 (PEG-silane, MW 459–591 g/mol) and N-trimethoxysilylpropyl-N,N,N trimethylammonium chloride (TA-silane, 50% in methanol) were purchased from Gelest (Morrisville, PA). 99.5% ethanol was purchased from Choneye Pure Chemicals. Dulbecco’s modified Eagle's medium (DMEM) was purchased from Gibco Co. Fetal bovine serum (FBS), penicillin-streptomycin (P/S), and trypsin were purchased from HyClone, GE. Fluorescein isothiocyanate–dextran (FITC-dextran, average mol wt 40,000) was purchased from Sigma-Aldrich. DAPI (4′,6-diamidino-2-phenylindole) was purchased from Thermo Fisher Scientific. Docetaxel (DTX) was obtained from ScinoPharm Taiwan Ltd. Temozolomide (TMZ) was obtained from MedChemExpress (Monmouth Junction, NJ, USA). Antibody CD-31 was purchased from Bioworld (BS1574). Secondary Goat anti-Rabbit IgG-FAM 488 was purchased from BioTnA (TAFB02-F). Cell Counting Kit 8 (WST-8/CCK8) was purchased from Abcam. FITC Annexin V Apoptosis Detection Kit with PI was purchased from BioLegend. Antiactive caspase 3 was purchased from Proteintech. A cleaved poly ADP-ribose polymerase (PARP) antibody was purchased from Cell Signaling Technology. VECTASTAIN Elite ABC-HRP Kit was purchased from Vector Laboratories. TUNEL assay was performed using the TUNEL Assay Kit HRP-DAB (ab206386) according to the manufacturer’s instructions.

### 2.2 Synthesis of Mesoporous Silica Nanoparticles with Various Ratios of TEOS to C8-Silane Modification on the Sidewall of Pores

The C8-MSN was synthesized by the method described in a previous study with modifications.^33,34^ Briefly, 0.29 g of CTAB was dissolved in 150 mL of ammonium hydroxide solution at 50 °C in a sealed beaker. After 15 min of stirring, 15.7, 39.2, 52.2, and 261 μL of the octyltriethoxysilane (the ratio of TEOS/C8-silane = 50:1, 20:1, 15:1, and 3:1) with 60, 150, 200, and 1000 μL of ethanol was added and stirred for 30 min. After that, 333 μL of TEOS in 1.132 mL of ethanol was added to the solution under vigorous stirring. After 1 h of stirring, another addition of 217 μL of TEOS in 0.868 mL of ethanol was added. After 3 h of the reaction, the PEG-silane (1000 μL) with TA-silane (155.8 μL) in 3.2 mL of ethanol was introduced into the reaction, and then 50 μL of TEOS in 300 μL of ethanol was immediately added. After stirring for 1 h, the mixture was aged at 50 °C without stirring overnight. Then, the solution was sealed and placed in an oven at 70 °C for 2 days of hydrothermal treatment. The as-synthesized sample was washed and collected by centrifugation. To remove the surfactant in the pores of the MSN, the as-synthesized sample was incubated in 50 mL of acidic ethanol containing 848 μL (first time) and 50 μL (second time) of hydrochloric acid (37%) for 1 h of extraction at 60 °C. The products were washed and harvested by centrifugation and finally stored in ethanol.

### 2.3 Characterization of MSN

Transmission electron microscopy (TEM) images were taken on a Hitachi H-7700 instrument with a 100 kV voltage. Sigma Scan Pro 5.0 software (Ashburn, VA) was used for the nanoparticle size distribution analysis. Dynamic light scattering (DLS) measurements of C8-MSN suspended in H_2_O and PBS buffer were performed by a Nano ZS90 laser particle analyzer (Malvern Instruments, U.K.). Zeta potentials of C8-MSN (0.1 mg mL^–1^) were measured in a diluted PBS solution. The N_2_ adsorption–desorption isotherms of the C8-MSN were obtained from a Micrometrics ASAP 2020 (Norcross, GA). The surface area and pore size were calculated using the Brunauer–Emmet–Teller (BET) equation and the standard Barrett–Joyner–Halenda (BJH) method.

### 2.4 Preparation of DTX@C8-MSN

Ten milligrams of C8-MSN were dispersed in 0.125 mL of H_2_O; 9.45 μL of DTX solution, prepared in DMSO at 50 mg mL^–1^, was slowly dropped into the particle solution with vigorous stirring. After being thoroughly mixed, the solution was diluted with H_2_O to decrease the DMSO concentration, and the trace amount of free DTX aggregates was removed by filtration through a 0.22 μm filter. Finally, the DTX@C8-MSN solution was washed with 7 to 10-fold water and stored at 4 °C.

### 2.5 DTX Quantification by High-Performance Liquid Chromatography (HPLC)

The loaded DTX amount of DTX@C8-MSN was analyzed by high-performance liquid chromatography. The 10 μL of DTX@C8-MSN stock solution (20 mg mL^–1^) was mixed with the 31 μL of H_2_O, 24 μL of aqueous HF (1.5%), and 65 μL of ACN. After 10 min of sonication at R.T., the solution was centrifuged at 6000 rpm for 10 min. The 120 μL of supernatant was taken for HPLC analysis (1260 Infinity II LC System, Agilent). The HPLC conditions were as follows: Sepax Bio-C18 column (4.6 mm × 250 mm, 5 μm of particle size), a flow rate of 1.0 mL min^–1^, linear 1% min^–1^ gradient from 48 to 38% A (solvent A, 99.9% water, 0.1% TFA; solvent B, 90% acetonitrile, 0.1%TFA), and a measured wavelength of 229 nm. The loading capacity is presented by the weight ratio of the DTX to the DTX@C8-MSN. The loading efficiency is presented by the weight ratio of the DTX loaded in C8-MSN to the total amount of DTX used for loading.

### 2.6 In Vitro Drug Release Assays

DTX from DTX@C8-MSN was assessed using a dialysis method. DTX@C8-MSN, suspended in 4 mL of PBS at a concentration of 0.25 mg DTX mL^–1^, and DTX, dissolved in PBS with 50% EtOH at a concentration of 0.25 mg mL^–1^, were separately introduced into the dialysis membrane made of regenerated cellulose (MWCO 12–14 kDa) and sealed with SpectraPor closures. The dialysis membrane was immersed into a 100 mL glass bottle containing 96 mL of the release medium (phosphate buffered solution (pH 7.4) with 2% Tween-80 and 10% EtOH) and a stir bar. The bottle was incubated in a water bath at 37 °C with continuous stirring. At a predetermined time, 300 μL of the release medium was collected for analysis and replaced with an equal volume of fresh release media. To quantify the released docetaxel amount in the release medium, 130 μL of the medium solution was taken for HPLC analysis.

### 2.7 Degradation Behavior of C8-MSN

To investigate the degradation behavior of C8-MSN, C8(15:1)-MSN-PEG/TA was dispersed in a PBS buffer solution (0.2 mg mL^–1^) and incubated at 37 °C for up to 7 days. The morphology, hydrodynamic size, and count rate of nanoparticles in the solution were obtained from TEM observation and DLS measurement on days 1, 2, and 7 after incubation.

### 2.8 Storage Stability of DTX@C8-MSN

The storage stability of DTX@C8-MSN at 4 °C in different solutions, including H_2_O, saline, and 5% dextrose for 3 months, was evaluated by TEM (synthetic diameter), DLS (hydrodynamic size), the concentration of DTX, entrapped DTX percentage, and the appearance of solutions.

### 2.9 Analysis of the Entrapped Docetaxel Ratio in DTX@C8-MSN Stock Solutions

To measure the ratio of loaded DTX amount to the total DTX amount (loaded DTX + free DTX), the aggregated free docetaxel (if any) was separated by centrifuging the DTX@C8-MSN solution at 6000 rpm for 10 min. Then, the supernatant (DTX loaded in C8-MSN) was taken for HPLC analysis. The entrapped DTX ratio was calculated by dividing the loaded DTX amount by the total DTX amount.

### 2.10 Cell Viability Assay

TMZ-resistant U87MG cells (U87MG-R) were grown in Dulbecco’s modified Eagle’s medium (DMEM) supplemented with 10% fetal bovine serum, 100 μg mL^–1^ penicillin/streptomycin and 100 μM TMZ. The cells were maintained at 37 °C in a humidified atmosphere containing 5% CO_2_. The cells were seeded in a 96-well plate at a density of 10,000 cells per well for 24 h. Cells were treated with 100 μL of various concentrations of DTX@C8-MSN, DTX (0, 2.5, 6.25, 12.5, 25, 100, or 200 ng of DTX mL^–1^), or C8-MSN (0, 60, 150, 300, 600, 1200, 2400, and 4800 ng of C8-MSN mL^–1^). After incubation for 48 h, the cell viability of U87MG-Rcells was evaluated using the Cell Counting Kit-8. For the control, cells were incubated in a culture medium. The absorbance of the blank solution (100 μL of CCK-8 reagent) was subtracted from the absorbance of the control and samples. All experiments were performed in triplicates. The cell viability was calculated according to the following formula:
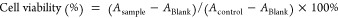


### 2.11 Hemolysis Assay

Mouse RBCs were isolated from whole blood samples collected from seven-week-old healthy BALB/c mice (BioLasco, Taiwan). The diluted RBC suspension was mixed with C8-MSN or DTX@C8-MSN solutions at various concentrations (1–1600 μg mL^–1^). Water and PBS solutions were incubated with RBC suspension as the positive (+) and negative (−) controls, respectively. All the samples were incubated at room temperature in the dark for 3 h and centrifuged, and the supernatant was taken to measure the absorbance (about 570 nm). The percent hemolysis of the RBCs was calculated using the formula:
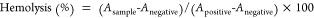


### 2.12 In Vitro Cell Uptake

U87MG-LUC cells were seeded in a 6-well plate at a density of 2 × 10^5^ cells per well and then treated with different concentrations of R-C8-MSN (250, 500, 750, and 1000 μg mL^–1^) for 24 h. The cell uptake of R-C8-MSN was analyzed by using flow cytometry.

### 2.13 In Vivo Biodistribution of R-C8-MSN in U87 Orthotopic Tumor-Bearing Mice

In vivo biodistribution images of R-C8-MSN were captured by using a fluorescence imaging instrument (IVIS, Lumina). Seven-week-old BALB/c nude mice, obtained from BioLASCO (Taiwan), were subcutaneously implanted with U87 glioma cells to establish an orthotopic xenograft tumor model. After a two-week period, the mice received an intravenous injection of R-C8-MSN at a dosage of 200 mg kg^–1^ through the tail vein. Following a 24 h injection period, the mice were euthanized, and major organs (heart, liver, spleen, lung, kidney, brain, and tumors) were excised for imaging. The fluorescence intensity was subsequently recorded by using an IVIS imaging system.

### 2.14 Apoptosis Detection Using Annexin V-FITC/PI Assay

According to the manufacturer’s instructions, cell apoptosis was quantified with Annexin V-FITC Apoptosis Detection Kit. Briefly, U87MG-LUC cells were placed on a 6-well plate at a density of 2 × 10^4^ per well and then treated with docetaxel (DTX, 50 ng mL^–1^), DTX@C8-MSN (equivalent to 50 ng mL^–1^ of DTX), and C8-MSN (equivalent to the concentration of DTX@C8-MSN) for 48 h. The total culture medium and cells were harvested, centrifuge-washed with cold PBS twice, and resuspended in 100 μL of AnnexinV binding buffer. After that, cells were stained with 5 μL of Annexin V-FITC and 10 μL of propidium iodide (PI) solution in the dark for 15 min at room temperature and added with another 400 μL of Annexin V binding buffer. The early apoptotic (Annexin V^+^/PI^–^) and late apoptotic (Annexin V^+^/PI^+^) cells were analyzed by using flow cytometry.

### 2.15 In Vivo Two-Photon Imaging

The circulation of rhodamine-labeled C8-MSN (R-C8-MSN) in the cerebrovasculature in mice was evaluated by in vivo two-photon microscopy. Seven-week-old healthy BALB/c mice (BioLasco, Taiwan) were anesthetized for the skull-removed craniotomy according to previous reports^35−37^ and intravenously injected with fluorescent R-C8-MSN at a dose level of 200 mg kg^–1^. The real-time images were taken with a two-photon microscope with an infrared (850 nm) laser and a 20X water-immersion objective. The time-lapse images were recorded at different time points following intravenous injection of R-C8-MSN.

The BBTB penetration and tumor-targeting capability of R-C8-MSN were assessed by in vivo two-photon microscopy in the orthotopic U87MG-LUC tumor model. BALB/c nude male mice (7 weeks old) were employed for tumor transplantation. Animals were anesthetized with zoletil (20–40 mg kg^–1^) and xylazine (5–10 mg kg^–1^) mixture solution and immobilized on a stereotactic frame during tumor inoculation. U87MG-LUC cells (3 × 10^5^ cells/3 μL of PBS) were injected into the cortex at 2 mm lateral, 1.5 mm posterior, and 2 mm ventral from the central bregma using stereotactic guidance and a microprocessor single syringe. Twenty days after tumor implantation, the mice were intravenously injected with R-C8-MSN at a dose level of 200 mg kg^–1^. One day after injection, mice were anesthetized for the skull-removed craniotomy and intravenously injected with 60 μL of 2.5% (w/v) FITC-dextran (green fluorescence) to visualize cerebral vessels. The images of cerebral vasculature were acquired using a two-photon microscope at a depth of 150 μm below the cortical surface. The mean fluorescence intensity of R-C8-MSN in the normal brain region and brain tumor region was quantified by measuring the fluorescence intensity in the regions of interest (ROIs).

### 2.16 Immunofluorescence Staining Analysis

The brain tissues were collected and fixed for frozen sections after in vivo two-photon imaging. In the normal mice model, the sections were stained with primary antibody against CD-31 (1:300) at 4 °C overnight and secondary Goat anti-Rabbit IgG-FAM 488 at room temperature for 1 h to visualize cerebral vessels (green). DAPI was used to localize the cellular nuclei. In the orthotopic U87MG-LUC tumor model, brain sections underwent DAPI staining to visualize the nuclei of brain cells and highlight the tumor region, identified as the hypercellular area exhibiting intense DAPI staining. Distribution images of R-C8-MSN in whole-brain sections were captured by using the ImageXpress Pico Automated Cell Imaging System. Enlarged regions were further observed by fluorescence microscopy.

### 2.17 Antitumor Activity of DTX@C8-MSN or DTX in the Orthotopic TMZ-Resistant GBM Mouse Model

TMZ-resistant U87MG cells (U87MG-R) were maintained in DMEM supplemented with 10% fetal bovine serum, 100 μg mL^–1^ streptomycin/penicillin, and 100 μM TMZ. The animal experiments are approved by the Institutional Animal Care and Use Committee (IACUC) of Taipei Medical University. The glioblastoma multiforme (GBM) cells known as T98G, which express O6-methylguanine-DNA methyltransferase (MGMT), inherently resist temozolomide (TMZ). Conversely, MGMT expression is absent in U87MG cells. We cultivated TMZ-resistant U87MG cells by subjecting them to TMZ over a six-month period, subsequently confirming their resistance via viability assays.^38−41^ MGMT, a renowned DNA repair protein, can eliminate TMZ-induced DNA methylation, thus conferring TMZ resistance. However, we observed that MGMT expression remained suppressed in our TMZ-resistant cells. This finding implies that GBM cells can adapt to TMZ-induced stress even in the absence of MGMT expression and that the characteristics of TMZ resistance might fade once TMZ is removed. Due to the lack of MGMT expression, the TMZ-resistant phenotype can only persist in the presence of TMZ. Our previous studies clearly described the experimental procedure.^38,41,42^ The characteristic of TMZ-resistant U87MG was demonstrated previously.^39,40^ Briefly, NOD.CB17-Prkdcscid/NcrCrl mice (9 weeks old) used in this experiment were purchased from BioLASCO Taiwan Co., Ltd. (Taipei, Taiwan). For TMZ-resistant GBM transplantation, U87MG-R cells (6 × 10^5^ cells/5 μL DMEM) were injected into the cortex at 2 mm lateral, 2 mm posterior, and 3 mm ventral from the central bregma using stereotactic guidance and microprocessor single syringe. DTX was dissolved in the solution containing 20% DMSO, 20% Tween 80, and 60% PBS. After tumor cell inoculation, the orthotopic tumor-bearing mice were intravenously injected with DTX or DTX@C8-MSN at the dose levels of 5 or 10 mg DTX kg^–1^ on days 5, 9, 13, 26, 30, and 34. For the control group, mice were injected with 100 μL PBS solution (containing 20% DMSO, 20% Tween-80, and 60% PBS). The body weight of mice was measured every 3–4 days, and the survival time was monitored up to the point of spontaneous death. The median survival time (MST) was calculated by the Kaplan–Meier method using Prism 9.4 software (GraphPad, US). Two mice from each group were sacrificed on day 20, and the brains were excised, then fixation using 4% paraformaldehyde and embedded by paraffin. Five-μm slices were stained using hematoxylin and eosin and also used for IHC analysis.

### 2.18 Immunohistochemical Staining Analysis

Paraffin-embedded slices were stained with the antiactive caspase 3 or cleaved poly ADP-ribose polymerase (PARP) antibody using VECTASTAIN Elite ABC-HRP Kit.

### 2.19 Pharmacokinetics of DTX@C8-MSN in Rats

Four male Sprague–Dawley rats received DTX@C8-MSN at 10 mg docetaxel/kg dose via a 10 min i.v. infusion. Approximately 250 μL of blood was collected via tail vein or cannula from rats after the end of infusion at various times; 30 μL of plasma was spiked with the internal standard solution (DTX-d9). One milliliter of methyl tertiary-butyl ether was added, vortex-mixed, and centrifuged at 3000 rpm for 10 min. The supernatant was collected and evaporated under nitrogen gas. The dried residue was resuspended in 150 μL of 50% methanol and 0.1% acetic acid. The solution was transferred to an autosampler vial for analysis by an LC–MS/MS method that was partially validated, including accuracy and precision, as well as the lower limit of quantification.

## 3 Results and Discussion

### 3.1 Synthesis and Characterization of C8-MSN-PEG/TA for Drug Delivery

**Figure 1:**
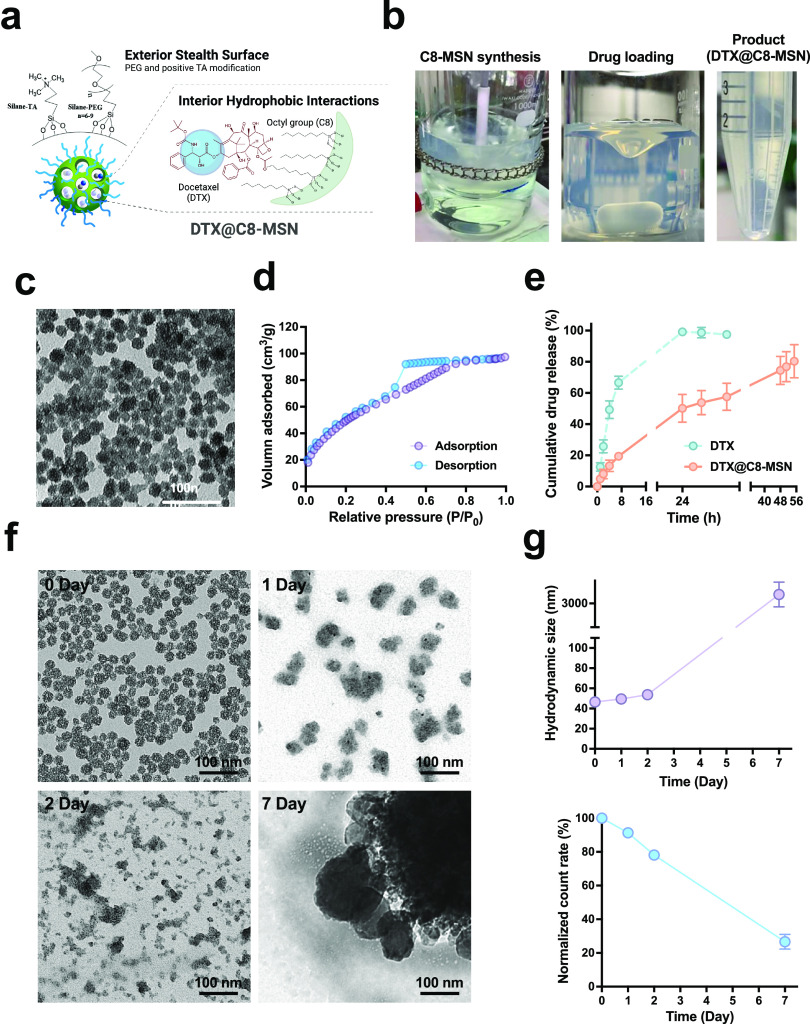
(a) Schematic diagram illustrates the design of the DTX@C8-MSN. The MSN was modified with trimethoxy (octyl) silane (C8-silane) on the sidewall of pores and functionalized short-chain PEG and quaternary ammonium group on the particle surface. (b) Appearance of the solution in different stages of the manufacturing process (C8-MSN synthesis, drug loading process, and purified product). (c) TEM images of the C8-MSN. (d) Nitrogen adsorption–desorption isotherms of C8-MSN were analyzed by a surface area and porosity analyzer. The surface area of C8-MSN was 194 m^2^ g^–1,^ and the average pore size was 1.68 nm. (e) In vitro drug release profiles of DTX and DTX-loaded C8-modified MSNs (DTX@C8-MSN) were analyzed in a release medium comprising PBS (pH 7.4) with 2% Tween-80 and 10% ethanol. (f) In vitro degradation of C8-MSN was incubated in PBS at 37 °C for days. The morphology of C8-MSN in PBS was observed by TEM. (g) Size and count rate of C8-MSN detected by DLS at a series of time points.

In this study, MSN with specific internal surface modification of pores and optimized external surface modification were synthesized based on a templated sol–gel process.^14^ The synthetic particle size of the MSN was controlled at approximately 30 nm. It has previously been reported that hydrophobic functional-silanes could be introduced as one of the silica sources in reaction to make the resulting MSN able to encapsulate hydrophobic molecules.^43^ However, introducing TEOS and hydrophobic functional silanes in a co-condensation approach during the nucleation and growth of the particles allows the functional groups to be dispersed throughout the particle, including their external surfaces. When multiple hydrophobic moieties were exposed on the surface of MSN, it would lead to particle aggregation in aqueous conditions due to the hydrophobic interaction between nanoparticles, thus not suitable for bioapplications, especially for injectable products. Therefore, a two-step co-condensation of different organo-silanes and TEOS was used to functionalize the inner and outer surfaces in MSN selectively. Triethoxy(octyl)silane (C8-silane) was used as a hydrophobic functional group and introduced at the beginning of the self-assembly stage; TEOS was added to form micelle/C8-silane/silicate clusters containing nuclei first, which was composed of a high percentage of C8-silane. Then, in the second step, more TEOS was added to start the growth process. This process made C8-silane primarily located at the internal pores of MSN. Also, a clear separation and growth period in synthesis leads to uniform sizes of the as-synthesized silica–surfactant nanoparticles. After the complete growth of the MSN, a mixture of mole ratio of PEG-silane/TA-silane (7:1) was added to modify the outer particle surface to enhance the aqueous dispersity and modulate the surface charge of MSN to neutral for better circulation and less nonspecific binding in the body.^44^ In addition, several molar ratios of TEOS/C8-silane (50:1, 20:1, 15:1, and 3:1) were adjusted to modulate the hydrophobic degree in the pores of MSN. The DTX loading capacity/loading efficiency of C8(20:1)-MSN-PEG/TA and C8(15:1)-MSN-PEG/TA was 4.30/93% and 4.41/95%, respectively, and the DTX loaded particles (DTX@C8(20:1)-MSN-PEG/TA and DTX@C8(15,1)-MSN-PEG/TA) had good dispersity in aqueous solutions and no significant difference in DLS sizes of particles before and after drug loading (Table S1). Hence, DTX was inferred to be loaded within the pores rather than attached to the outer surface of MSN. The resulting structure of DTX@C8-MSN is shown in Figure 1a.

Increasing the C8-silane proportion to a TEOS:C8-silane ratio of 3:1 caused severe particle aggregation in PBS, resulting in a DLS size of about 1 μm. In contrast, C8(50:1)-MSN-PEG/TA had poor DTX loading capacity due to insufficient C8-silane modification. This suggests that the C8 functional groups on the particle surface greatly impact particle dispersity (Table S1). C8(15:1)-MSN-PEG/TA was then selected for the mouse model studies due to its higher drug loading capacity, efficiency, and good dispersity (hereafter denoted simply as C8-MSN). Figure 1b shows the reactor and appearance of the suspension solution of DTX@C8-MSN. It appears to be a stable, transparent solution with slight light scattering. The TEM image and dynamic light scattering (DLS) data revealed uniform particle size and good dispersity in aqueous solutions of C8-MSN. The TEM diameter and hydrodynamic diameters (DLS) are about 29 ± 1.5 and 44.5 ± 1.6 nm (Figure 1c and Table S1). Nitrogen adsorption/desorption isotherms characterized the surface areas and pore size. The surface area was 194 m^2^ g^–1^, as calculated by the Brunauer-Emmet-Teller (BET) method and the average pore size was about 1.68 nm, determined by the Barrett–Joyner–Halenda (BJH) method (Figure 1d). Typically, unfunctionalized bare MSN would give a BET surface area above 1000 m^2^ g^–1^ and a pore diameter of 2.4 nm.^13^ Compared to bare MSN, the lower surface area and smaller pore size of C8-MSN indicated that the pore walls had been modified with C8-silane.

A reproducible and scalable manufacturing process is critical for translating nanomedicines from the bench to the industrial scale. C8-MSN exhibited excellent reproducibility and consistency in particle properties in different batches; the characteristics were analyzed from more than 5 batches, and the coefficient of variation of the particle size distribution (synthetic diameter and DLS size) was less than 10%, indicating consistent batch-to-batch reproducibility in production. Moreover, the DTX@C8-MSN possessed long-term stability in aqueous solutions, including IV injectable solutions such as saline and 5% dextrose, at 4 °C for more than 3 months. No significant difference in TEM sizes, DLS sizes, DTX concentrations, and entrapped DTX ratios was observed after three months of storage (Table S2). The stability of DTX@C8-MSN under long storage is important for future clinical study. The DTX was stably entrapped in the pores of C8-MSN at low temperatures but exhibited a slow-release property in vitro under simulated physiological conditions (37 °C in PBS), as shown in Figure 1e. Notably, DTX exhibited a fast-release characteristic, achieving approximately 99% release within 24 h. In comparison, the sustained release of DTX from C8-MSN extended over 50 h, culminating in a complete release. The DTX shows sustained release from C8-MSN over about 48 h to reach complete release. The DTX release rate is associated with the degradation of C8-MSN, which is hydrolyzed and degraded in aqueous media. The degradation proceeds from the inner or outer surface of the MSN, the interface between MSN and medium.^45^ Once the C8-functional moiety is hydrolyzed and dissociated from the particle, the adsorption force between C8-MSN and DTX diminishes, resulting in DTX release. The degradation process and rate of C8-MSN were investigated by incubating MSN in PBS at 37 °C for 7 days. The morphology, mesostructure, and size of C8-MSN were detected by TEM and DLS at a series of time points (Figure 1f,g). The degree of degradation gradually increased over time, and the porous structure of C8-MSN appeared unclear 1 day after incubation, which meant that the degradation proceeded on the periodic mesochannels. On day 2, particle aggregation was observed in the TEM images and DLS results, which inferred that some PEG molecules on the surface were detached due to degradation on the surface. Furthermore, almost all the C8-MSN were degraded and exhibited severe aggregation on day 7. The count rate in DLS measurements, defined as the number of photons detected per second, is directly related to the concentration of the nanoparticles in a sample. Therefore, monitoring the count rate over time can provide information about the stability or degradation of the nanoparticles in a solution. Here, the count rate of measured C8-MSN showed a gradual decline during the first day of incubation, followed by a rapid decrease until the final measurement. It indicated that C8-MSN was biodegradable and almost completely degraded within 7 days. Concurrently, the degradation of DTX@C8-MSN was accompanied by a slow drug release behavior, which would help lower toxicity compared with free DTX released as a bolus.

### 3.2 Assessment of the Cellular Uptake, Hemolytic Activity, and the Antiproliferative Effects of C8-MSN and DTX@C8-MSN

**Figure 2:**
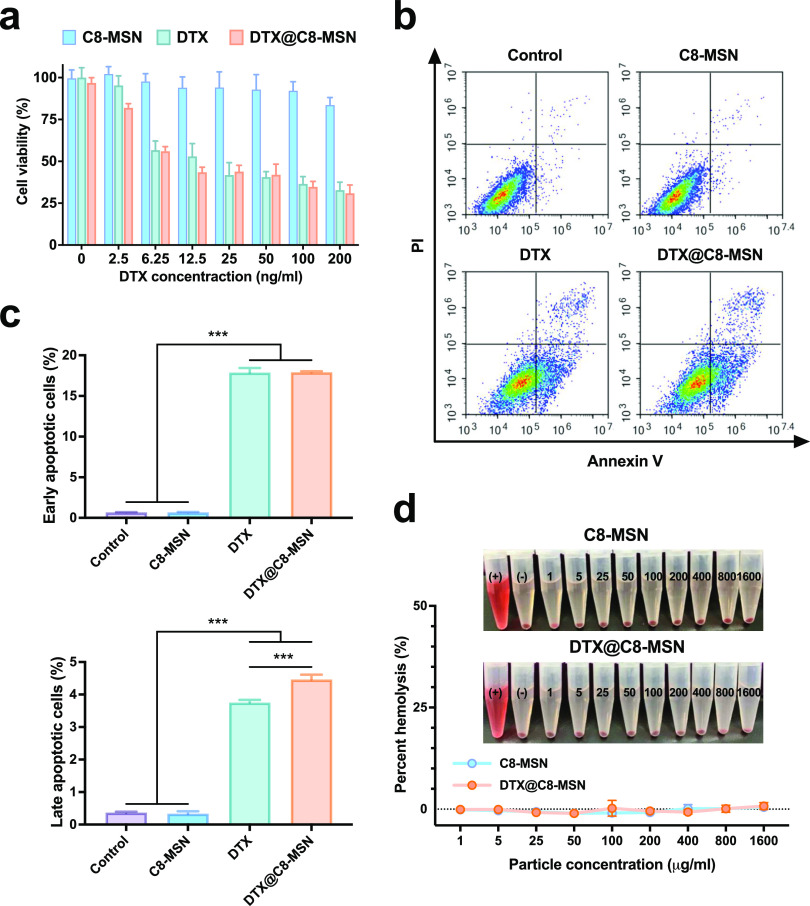
(a) Cell viability of DTX, C8-MSN, or DTX@C8-MSN in TMZ-resistant U87MG cells at different concentrations for 48 h. (b,c) Evaluation of apoptosis by the Annexin V/PI detection kit following the treatment of U87MG-LUC cells with DTX (50 ng mL^–1^), DTX@C8-MSN (equivalent to 50 ng mL^–1^ of DTX), and C8-MSN (equivalent to the concentration of DTX@C8-MSN) for 48 h. **p < 0.01, ***p < 0.001. (d) Hemolysis assays for C8-MSN and DTX@C8-MSN. RBCs were incubated in different concentrations of C8-MSN or DTX@C8-MSN (ranging from 1 to 1600 μg mL^–1^) for 3 h. The hemolysis was quantified by measuring the hemoglobin released from hemolyzed RBCs in the supernatant. Water (+) and PBS (−) are positive and negative controls, respectively. Data represent the mean of at least three independent experiments.

To investigate the cellular internalization of C8-MSN, the uptake of RITC conjugated C8-MSN (R-C8-MSN) was examined by using flow cytometry in human malignant glioblastoma cells (U87MG-LUC cells). Results showed that higher doses of C8-MSN led to increased cellular uptake (Figure S1). To further evaluate the DTX-induced cytotoxicity, as shown in Figure 2a, the cell viability of DTX, C8-MSN, and DTX@C8-MSN in TMZ-resistant U87MG cells was performed using Cell Counting Kit-8. DTX and DTX@C8-MSN could significantly induce cell death in a concentration-dependent manner. The IC50 value of DTX@C8-MSN (17.8 ng mL^–1^) was slightly better than that of DTX (22.0 ng mL^–1^), possibly due to the enhanced delivery of DTX by C8-MSN. C8-MSN alone had no cellular toxicity, revealing its biocompatibility.

To confirm the mechanism of action on cell death, the apoptotic cells were detected using an Annexin V/PI detection kit and quantitatively analyzed by flow cytometry (Figures 2b,c and S2). As compared to the control and C8-MSN, cells treated with DTX@C8-MSN (equivalent to 50 ng mL^–1^ of DTX) significantly increased in the early apoptosis (Annexin V positive/PI negative, 17.91 ± 0.12%), which was similar to 50 ng mL^–1^ of DTX (17.87 ± 0.57%). It was noticed that the late apoptosis (Annexin V positive/PI positive) of DTX@C8-MSN treatment (4.46 ± 0.15%) presented higher than that of DTX (3.75 ± 0.08%), suggesting that DTX could be better delivered into cells by C8-MSN. Changes in cell morphology during apoptosis, such as shrinkage of the cells, were also observed (Figure S2). Taken together, the results demonstrated that DTX@C8-MSN displayed therapeutic potential, enabling the proliferation inhibition of U87MG cells via enhanced cellular uptake for DTX delivery, followed by cell apoptosis.

The hemolysis study demonstrated that C8-MSN and DTX@C8-MSN had negligible to no hemolysis effects at all of the tested concentrations (from 1 to 1600 ng mL^–1^) (Figure 2d), which indicated that C8-MSN did not affect the membrane integrity of RBCs. DTX@C8-MSN would be more suitable and safer for intravenous injection than the current formulation of docetaxel (Taxotere), which contained a high concentration of nonionic surfactant Tween 80 that would cause severe hemolysis.^46,47^

### 3.3 Blood Circulation and Tumor Penetration of C8-MSN for Brain Tumor Targeting

**Figure 3:**
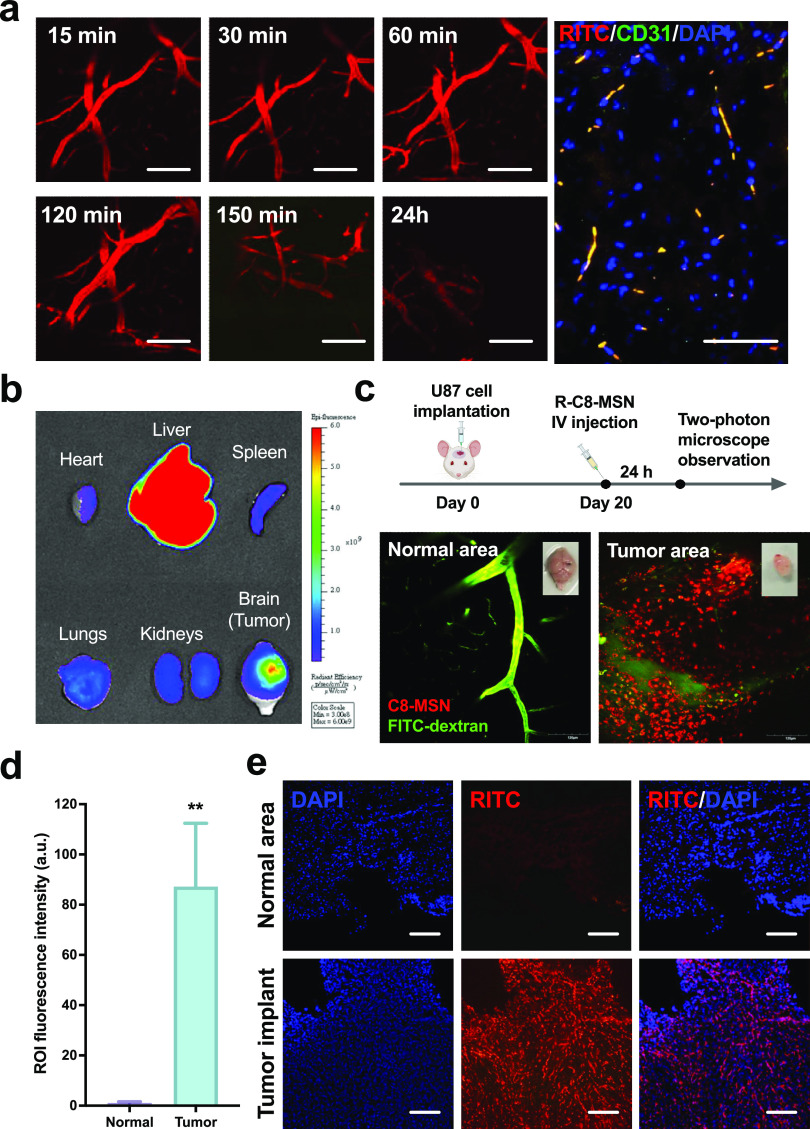
(a) Two-photon time-lapse imaging of R-C8-MSN in cerebrovasculatures of mice at different time points after injection (scale bar, 120 μm). After 24 h postinjection, the brain tissues were excised and fixed for frozen sections. The sections were immunofluorescently stained with red, green, and blue signals representing R-C8-MSN, Alexa Fluor 488-stained CD31, and DAPI-stained cell nuclei, respectively (Scale bar, 70 μm). (b) Biodistribution imaging was conducted on orthotopic brain tumor-bearing mice using R-C8-MSN, and the results were obtained from the in vivo imaging system (IVIS). (c) Schematic diagram of the experimental design (bottom panel). In vivo two-photon microscopy images of R-C8-MSN (red) in the cerebral vasculature of orthotopic tumor-bearing mice were acquired 24 h after injection. The blood vessels were visualized by IV injection of dextran-FITC (green). The tissues of the normal brain and the tumor excised from the cerebral right hemisphere were depicted in the upper right box. (d) Quantification of mean fluorescence intensity of R-C8-MSN in ROIs in the normal brain region and brain tumor region was calculated by ImageJ software (details are shown in Figure S3) and presented as mean ± SD (*n* = 3), **p < 0.01. (e) Histological examination of the nontumor region and tumor region was performed to assess the tumor-targeting capability of R-C8-MSN. R-C8-MSN was shown in red and the nuclei (DAPI) in blue (Scale bar, 200 μm).

To assess the blood circulation of C8-MSN, we administered R-C8-MSN intravenously in mice and used two-photon imaging techniques to evaluate its circulation in the bloodstream. R-C8-MSN particles (red fluorescence signals) were noticeably observed in the cerebral vessels within the first 2 h after injection, followed by a gradual decline over time (Figure 3a). Even after 24 h postinjection, R-C8-MSN particles were still observed circulating in the blood vessels, indicating that the reticuloendothelial systems (RES) would not scavenge R-C8-MSN particles out of circulation rapidly.^48^ To further validate the localization of C8-MSN in the cerebral vasculature, the brain sections were stained with anti-CD-31 (green) and DAPI (blue) to visualize the blood vessels and cell nuclei. The colocalization of R-C8-MSN fluorescence (red) with CD31 staining (green) revealed that nanoparticles were circulated inside vessels.

To explore the capability of R-C8-MSN as a carrier for delivering anticancer drugs into brain tumors, we aim to overcome the limitations of blood–brain tumor barrier (BBTB) penetration. In vivo verification of drug distribution was conducted by using orthotopic brain tumors. IVIS imaging clearly depicted the accumulation of R-C8-MSN in the liver tissue, as illustrated in Figure 3b. Remarkably, despite the substantial accumulation in the liver, the tumor tissue exhibited a strong signal, suggesting that R-C8-MSN has the potential to cross the BBTB and target tumors with a significant EPR effect. Solid tumors are often surrounded by various cell types and components such as stromal fibroblasts and collagen, making it challenging to deliver nanoparticles into the core of the tumors. Previously, we investigated the physicochemical properties of MSNs and their penetration behavior in spheroids by two-photon microscopy.^49^ Superior tumor penetration was observed in the small-sized MSN (∼30 nm) with external modification with PEG and quaternary ammonium groups. The results provide significant implications for the design of C8-MSN. In this study, we employed an orthotopic GBM xenograft model (intracranial implantation of U87MG-LUC cells in mice) to verify the effectiveness of C8-MSN in penetrating the blood–brain tumor barrier (BBTB) and targeting tumors. One day after intravenous injection of C8-MSN in tumor-bearing mice, the particle distribution in both normal brain regions and the brain tumor was examined by two-photon fluorescence microscopy. In Movie S1, clear differences are observed in the vascular patterns between the tumor site and normal tissue. Deep in vivo imaging shows a higher concentration of microvessels clustered within the tumor area characterized by their irregular and uneven distribution. C8-MSN (displayed in red) filled the perivascular space surrounding blood vessels (green) in the brain tumor region, indicating that R-C8-MSNs crossed the BBTB and diffused into the tissue. However, no R-C8-MSN signals were observed in the normal brain region outside the cerebral vessels. The fluorescence intensity of R-C8-MSN in the tumor site was much higher than in the normal brain area (Figures 3c,d and S2). The brain sections were stained with DAPI, which binds to DNA and serves as a marker for cell nuclei. This staining was performed to validate the tumor-targeting capability of R-C8-MSN. The distribution of R-C8-MSN in whole-brain imaging is depicted in Figure S4. The tumor region was identified by its hypercellular area, characterized by dense DAPI staining compared to that of the normal region. The enlarged images of histological sections of the brain, as shown in Figure 3e, revealed that the majority of R-C8-MSN particles accumulated in the tumor region, marked by intense DAPI staining. Rare nanoparticles were observed in healthy brain areas. In summary, the findings suggested that C8-MSN crosses the BBTB and is effectively targeted to brain tumors.

### 3.4 DTX@C8-MSN As a Promising Therapy for TMZ Refractory GBM Treatment

**Figure 4:**
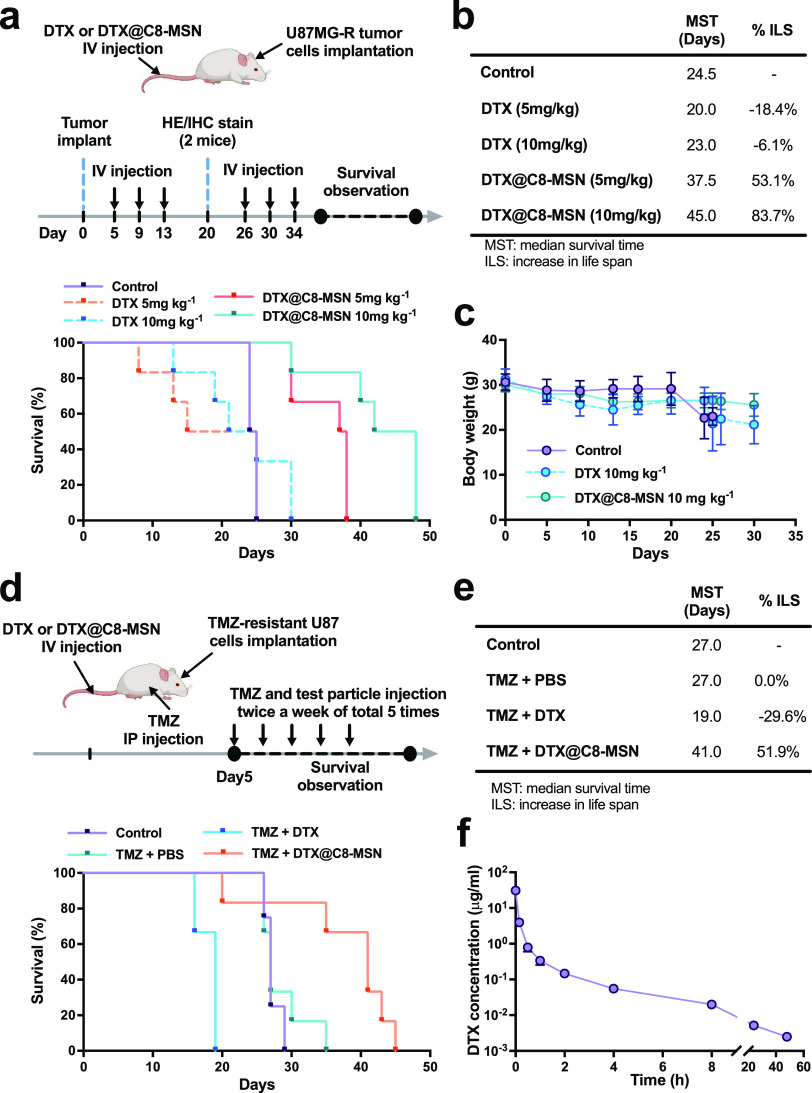
(a) Survival experiment of DTX or DTX@C8-MSN in orthotopic U87MG-R tumor-bearing mice, which received intravenous injections of DTX or DTX@C8-MSN at the dose levels 5 or 10 mg DTX kg^–1^ for a total of six administrations, respectively. Kaplan–Meier plots were used to display the survival rate. (b) Summary of the median survival time (MST) and the percentage of increase in life span (%ILS). (c) Change in the body weight of the control group and mice treated with DTX or DTX@C8-MSN at the dose of 10 mg DTX kg^–1^ was presented as mean body weight ± SD. (d) Orthotopic U87MG-R tumor-bearing mice were intravenously injected with DTX or DTX@C8-MSN at a dose of 10 mg DTX kg^–1^ in combination with IP administration of TMZ (10 mg kg^–1^). Kaplan–Meier plots were used to display the differential survival rate of the different treatment groups. (e) Summary of the median survival time (MST) and percentage of increase in life span (%ILS) for each group. (f) Plasma concentration–time profiles of docetaxel, including free and loaded forms, in SD rats (*n* = 4) after the i.v. administration of DTX@C8-MSN at the dose of 10 mg DTX kg^–1^.

TMZ is regarded as a first-line chemotherapeutic in GBM treatment. Unfortunately, over 50% of GBM patients receiving TMZ therapy do not respond to treatment and exhibit drug resistance. Effective chemotherapy for TMZ refractory GBM treatment is sorely needed.^50^ The orthotopic TMZ-resistant GBM (U87MG-R) xenografts in mice were established to evaluate and compare the efficacy of DTX or DTX@C8-MSN. Orthotopic U87MG-R tumor-bearing mice were treated with DTX or DTX@C8-MSN at dose levels of 5 or 10 mg of DTX kg^–1^. The mice treated with DTX@C8-MSN were noted to have effective tumor inhibition and significantly longer overall survival compared with the control and DTX groups (Figure 4a). The DTX@C8-MSN increased the median survival time in a dose-dependent manner, and the percentage of increase in life span of the DTX@C8-MSN groups at the dose of 5 or 10 mg DTX kg^–1^ over that of the PBS group was 53.1 or 83.7%, respectively (Figure 4b). In contrast, the mice treated with DTX revealed a median survival time similar to or shorter than that of the control group, indicating that DTX likely had some systemic toxicity while being ineffective against brain tumors. In addition, DTX@C8-MSN had lower toxicity based on less weight loss than DTX (Figure 4c). The therapeutic effect of DTX or DTX@C8-MSN in combination with TMZ was also evaluated, as shown in Figure 4d. Five days after U87MG-R cells implantation, the orthotopic tumor-bearing mice were intravenously injected with DTX or DTX@C8-MSN at 10 mg DTX kg^–1^ in combination with TMZ (10 mg kg^–1^, IP injection) twice per week for a total of 5 administrations. The survival rate was recorded up to spontaneous death, and the median survival time (MST) was calculated using the Kaplan–Meier method. Compared to the control group, TMZ could not extend the survival period in this model. In contrast to the DTX group causing adverse effects, DTX@C8-MSN significantly extends the MST from 19.0 to 41.0 days (Figure 4e). These results demonstrated that DTX@C8-MSN, used either alone or in combination with TMZ, significantly reduced tumor size, adverse effects, and prolonged survival.

In addition, we examined the pharmacokinetics (PK) of DTX@C8-MSN in rats after a single administration of DTX@C8-MSN via a 10 min IV infusion. Table 1 briefly summarizes the PK parameters of DTX formulations in this work (Figure 4f) and previous studies. In comparison to the solvent-based DTX formulation (Taxotere),^51^ the rats administered DTX@C8-MSN exhibited higher DTX plasma levels and areas under the concentration–time curve (AUC), as well as a slower clearance rate (CL) and decreased volume of distribution (Vz). The same trend was observed when treating rats with polymer-based DTX formulations, such as DTX@PLA–PEG and DTX@PLA/PLGA–PEG.^51^ Notably, the half-life (*t*_1/2_) varied according to the composition and physicochemical properties of the NP formulations. While an extended circulatory presence of NP formulations may increase their accumulation at the target site and benefit pharmacodynamics (PD) efficacy, several studies have shown that augmentation of the systemic presence of drugs does not always result in better therapeutic outcomes.^52,53^ Our research revealed that the enhanced penetration, internalization, and subsequent drug release of DTX@C8-MSN in tumors, in combination with an appropriate PK profile, led to an improvement in the therapeutic index of DTX.

**Table 1:** Pharmacokinetic Parameters of Various DTX Formulations^a^

		DTX@C8-MSN	taxotere	DTX@PLA–PEG	DTX@PLA/PLGA–PEG
parameters	unit	silica-based DTX formulation	solvent-based DTX formulation	polymer-based DTX formulation	polymer-based DTX formulation
dose	mg kg^–1^	10	5	5	5
Cmax	μg mL^–1^	30.8 ± 5.44	0.595	67.3	19.6
AUC_0-∞_	h μg mL^–1^	7.4 ± 0.839	0.489	327.7	22.6
*t*_1/2_	h	8.02 ± 3.49	5.2	6.6	1.9
CL	L h^–1^ kg^–1^	1.36 ± 0.149	9.289	0.011	0.237
Vz	L kg^–1^	15.7 ± 6.59	70.4	0.103	0.665

a Parameters of taxotere, DTX@PLA–PEG, and DTX@PLA/PLGA–PEG are obtained from a previous rat study.^50^

**Figure 5:**
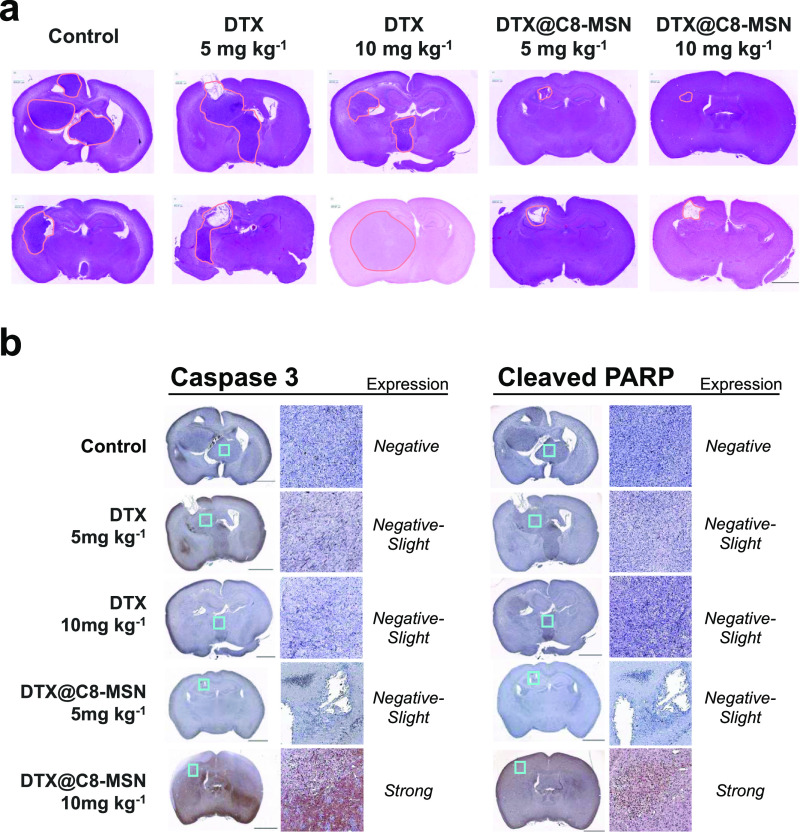
(a) HE staining images of brain tissues were obtained from mice on Day 20 after tumor implantation, as described in Figure 4a. The edge of the tumor area, diagnosed via histopathological analysis with HE staining, was marked in orange. (b) Immunohistochemical (IHC) staining for paraffin sections of brain tissues using the antibody targeting active caspase 3 or cleaved PARP.

To assess and compare the tumor size in different experimental groups, two mice from each group were sacrificed on day 20 (the experimental diagram is described in Figure 4a), and the brains were collected for histological examination. As shown in Figure 5a, histological staining revealed that the tumor size in mice administrated with DTX@C8-MSN was smaller than that of control and DTX groups, and DTX did not reduce the tumor size. In addition, the expression levels of apoptosis biomarkers, including caspase 3 and cleaved PARP, in brain tissues were conducted to evaluate the DTX-induced apoptosis. DTX@C8-MSN (at a dose of 10 mg kg^–1^) induced apoptosis characterized by increasing levels of active caspase 3 and cleaved PARP (Figure 5b). The results demonstrated that DTX was delivered by C8-MSN into brain tumor tissues and induced cancer cell death, resulting in decreased tumor size and improved survival time. Treatment with DTX@MSN alone is sufficient to inhibit the growth of GBM and extend the survival period of experimental mice. TUNEL assay revealed that DTX@MSN obviously increased the apoptotic cells inside the tumor (Figure S5).

TMZ has been used as the standard chemotherapy for malignant glioma since 2005. However, drug resistance invariably occurs. To date, various kinds of clinical trials have been performed for GBM with novel drugs. However, no drugs exceeded the effect of TMZ because of difficulties in crossing the BBTB. This study shows that a small MSN as a nanocarrier can overcome the BBTB problem. This opens the possibility of MSN carrying other potential drug candidates for brain tumors.^54^

In summary, we have developed a nanocarrier C8-MSN with specific modifications on the outer surfaces and in pore walls, enabling the hydrophobic DTX to load efficiently without affecting the dispersibility of the particles. DTX@C8-MSN had the capabilities of delivering DTX across the BBTB and targeting brain tumors, which resulted in an effective reduction in the tumor size, mitigated drug-related toxicity, and significantly prolonged survival compared to treatment with free DTX.

## 4 Conclusions

We have developed a customized MSN with a small TEM size (30 nm) with properties that allow for an injectable nanoformulation with DTX. The internal pore surfaces of the MSN are functionalized with a C8 moiety to enhance DTX water solubility (approximately a thousand times improvement, from μg mL^–1^ to mg mL^–1^) without using detergents—which have been implicated in systemic adverse reactions with the current DTX formulation in clinical use. The external surface of MSN was functionalized with PEG and organic quaternary ammonium to increase BBTB penetration in order to enhance tumor-targeting of DTX for the treatment of glioblastoma multiforme. The MSN delivery system also diminished the drug-related adverse effects that occur with free DTX.

The encouraging results with DTX@C8-MSN suggest that it could be developed as a clinical drug since it has (a) good drug loading and delivery profile, (b) excellent targeting and BBTB penetration, and (c) scalability of manufacture and stability of the drug during storage.

Tumor metastasis and recurrence continue to be considerable challenges in curing cancers. Due to the difficulties in the complete removal of the primary tumor in surgeries and the poor drug delivery efficiency of chemotherapy, brain tumor metastasis often occurs when the primary tumor is a lung or breast. Because the brain tumor sites tend to be dispersed, surgery is often not an option and the prognosis is usually very poor. Our C8-MSN with encapsulated DTX may overcome this problem because it can efficiently penetrate BBTB and accumulate at tumor sites to effectively inhibit the proliferation of metastasized tumors. Finally, the nanocarrier C8-MSN may also make the delivery of other hydrophobic chemotherapeutic agents across the BBTB possible. MSN-based delivery systems have the potential to expand the utility of FDA-approved drugs and dramatically increase their safety and efficacy.

## Supporting Information Available

Characteristics of MSN with various ratios of C8-silane modification; stability tests of DTX@C8-MSN; cellular uptake of C8-MSN; changes in cell morphology following treatments of C8-MSN, DTX and DTX@C8-MSN; regions selected for quantitative analysis of fluorescence images in Figure 3; distribution of R-C8-MSN in brain sections contrasting normal and tumor tissues post intravenous administration; TUNEL staining in GBM sections to identify apoptotic cells; and in vivo two-photon image stacks of the brain and tumor region (PDF)
Differences in the vascular patterns between the tumor site and normal tissue (MPG)

## Supplementary Material

am4c04289_si_001.pdf

am4c04289_si_002.mpg

## Notes

The animal studies addressed in this paper were ethically reviewed and approved by Taipei Medical University, Laboratory Animal Center (LAC), under the Institution of Animal Care Use Committee (license ID: LAC-2018–0164).

## Notes

The authors have given their consent for the manuscript to be published in Journal of Nanobiotechnology
