# Supplementary material for: Overcoming the Blood–Brain Tumor Barrier with Docetaxel-Loaded Mesoporous Silica Nanoparticles for Treatment of Temozolomide-Resistant Glioblastoma
Source: ACS Appl Mater Interfaces. 2024 Apr 17;16(17):21722–35. doi: 10.1021/acsami.4c04289 (PMC11071047; doi:10.1021/acsami.4c04289)
Supplement: Supplementary file 1 — am4c04289_si_001.pdf [file am4c04289_si_001.pdf]

# Supporting Information

## Overcoming the Blood-Brain Tumor Barrier with Docetaxel-Loaded Mesoporous Silica Nanoparticles for Treatment of Temozolomide-Resistant Glioblastoma

Tsung-I Hsu, Yi-Ping Chen, Rong-Lin Zhang, Zih-An Chen, Cheng-Hsun Wu, Wen-Chang Chang, Chung-Yuan Mou, Hardy Wai-Hong Chan, Si-Han Wu*

**Table S1.** The characteristics of MSN with various ratios of C8-silane modification.

| The ratios of TEOS/ C8-silane | Ave. Size (nm) (TEM) | DLS (d, nm) / PDI | | | DTX Loading Amount | DTX Loading Efficiency |
|---|---|---|---|---|---|---|
| | | In water | in PBS | | | |
| | | | - | With DTX | | |
| 50:1 | 32.5 ± 6.6 | 57.8 / 0.052 | 56.4 / 0.060 | 55.9 / 0.034 | 0.003% | ~0% |
| 20:1 | 33.4 ± 6.0 | 47.7 / 0.086 | 48.4 / 0.090 | 48.3 / 0.077 | 4.30% | 93% |
| 15:1 | 29.0 ± 4.6 | 43.1 / 0.118 | 43.8 / 0.073 | 44.5 / 0.086 | 4.41% | 95% |
| 3:1 | NA | 131.3 / 0.439 | 945 / 0.488 | NA | NA | NA |

**Table S2.** Stability analysis of DTX@C8-MSN after 3 months of storage

| Storage Medium | TEM size (nm) | DLS size (d.nm)/PDI | DTX conc. (mg/mL) | Entrapped DTX (%) | Appearance State |
|---|---|---|---|---|---|
| $H_2O$ (initial) | 26.0 ± 3.1 | 41.9 / 0.146 (in $H_2O$)<br>42.0 / 0.068 (in PBS) | 6.24 | ~99% | Clear |
| $H_2O$ (3 months) | 26.2 ± 3.0 | 41.6 / 0.152 (in $H_2O$)<br>40.6 / 0.084 (in PBS) | 6.81* | ~99% | Clear |
| Saline (initial) | 28.5 ± 5.6 | 47.1 / 0.079 (in $H_2O$)<br>48.7 / 0.081 (in PBS) | 6.52 | ~99% | Clear |
| Saline (3 months) | 27.4 ± 4.9 | 47.0 / 0.080 (in $H_2O$)<br>48.0 / 0.103 (in PBS) | 6.74* | ~99% | Clear |
| 5% Dextrose (initial) | 29.6 ± 4.3 | 47.0 / 0.135 (in $H_2O$)<br>47.3 / 0.082 (in PBS) | 6.63 | ~99% | Clear |
| 5% Dextrose (3 months) | 26.5 ± 5.3 | 46.1 / 0.151 (in $H_2O$)<br>47.1 / 0.084 (in PBS) | 6.66 | ~99% | Clear |

* The observed increase in DTX concentration could be attributed to the slight solvent evaporation

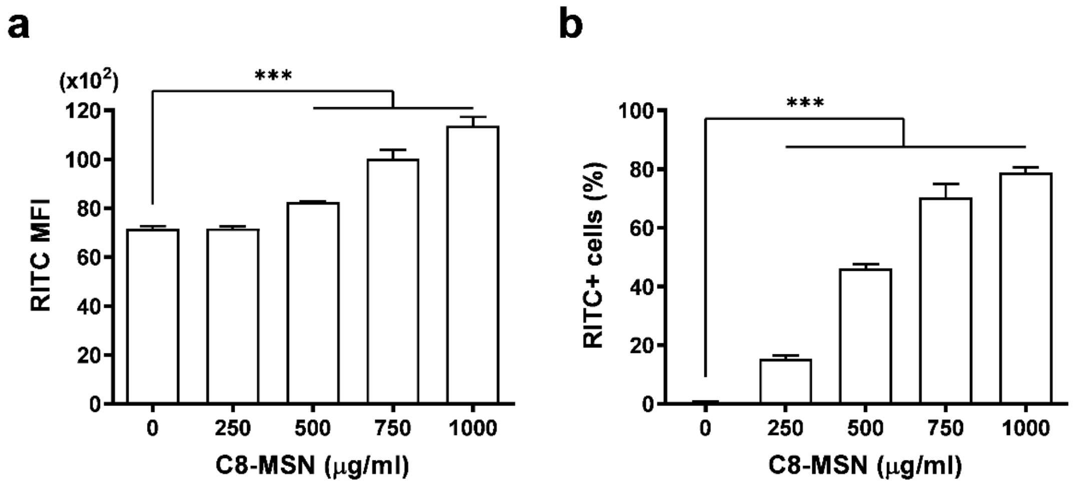


**Figure S1.** The cellular uptake of RITC conjugated C8-MSN at different concentrations in U87MG-LUC cells was measured by flow cytometry after treatment for 24 hours. (a) MFI and (b) percentage of positive cells.

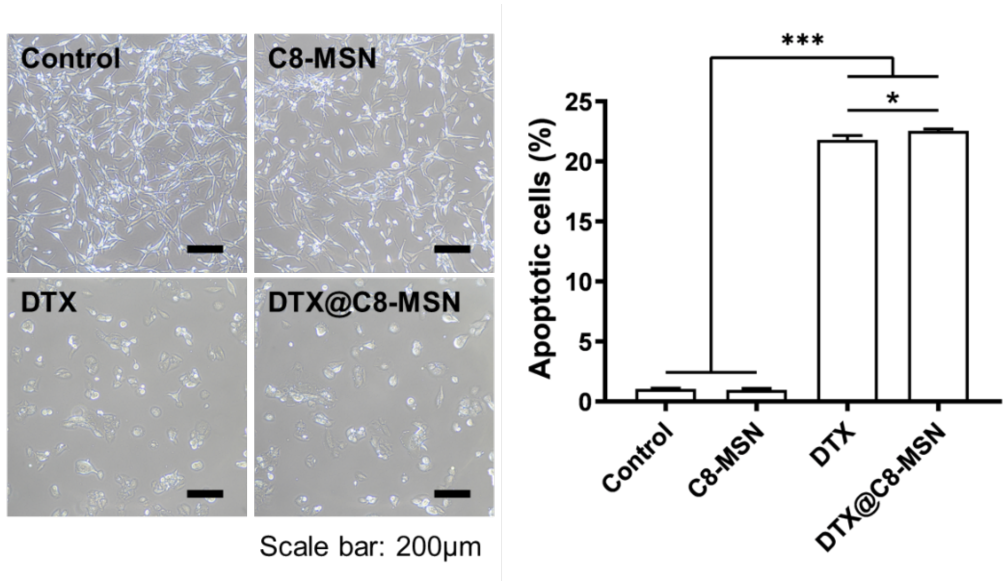


**Figure S2.** Changes in cell morphology and quantitative analysis of apoptosis following treatments of C8-MSN, DTX and DTX@C8-MSN, respectively.

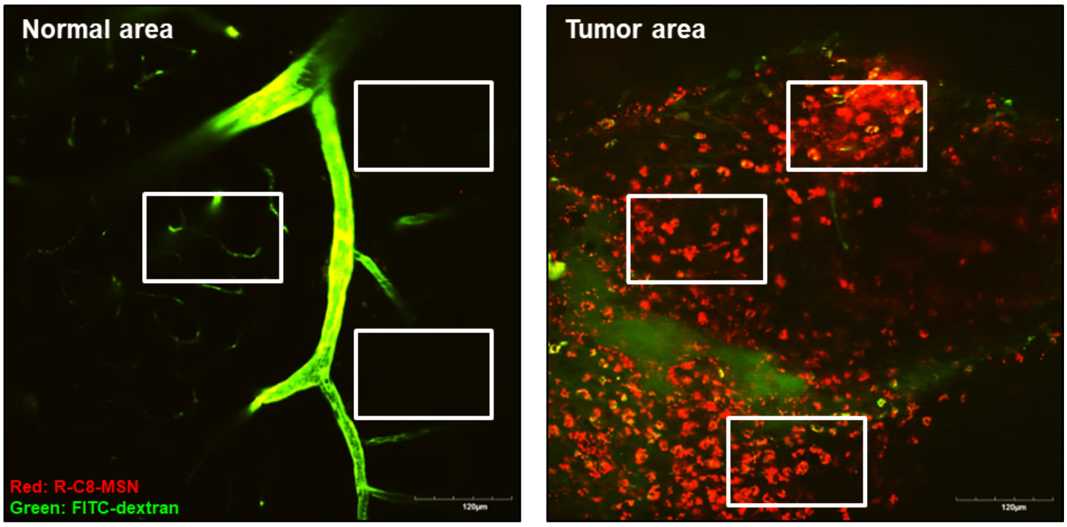


**Figure S3.** Quantitative analysis of fluorescence intensity in regions of interest (ROI) performed on three different regions in two-photon microscopy images, as shown in Figure 3.

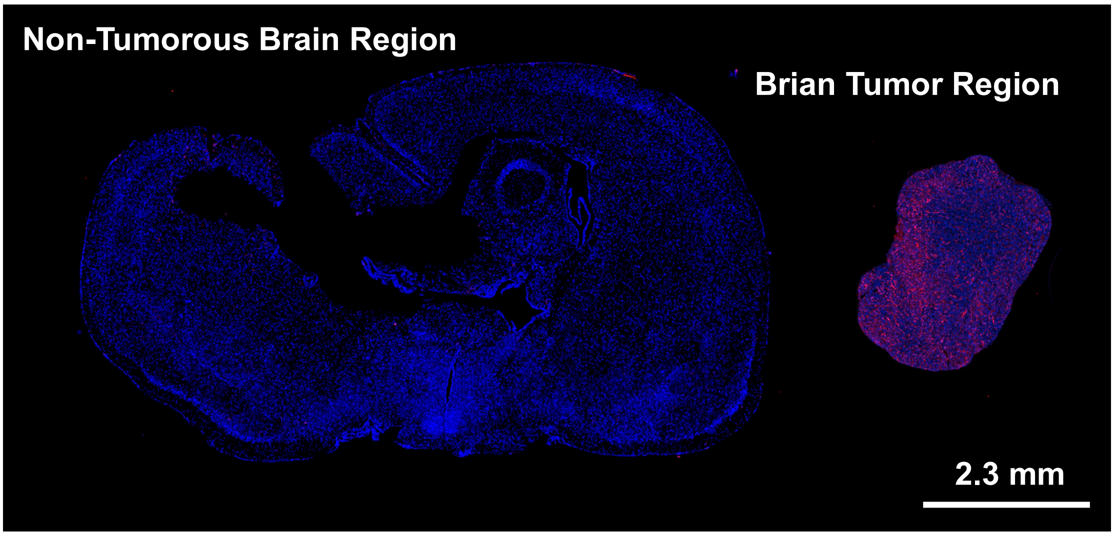


**Figure S4**. Distribution of R-C8-MSN in whole brain sections 24 hours after intravenous administration, as imaged using the ImageXpress® Pico Automated Cell Imaging System (Scale bar, 2.3 mm). The left panel depicts normal brain tissue, while the right panel shows tumor tissue from the same brain.

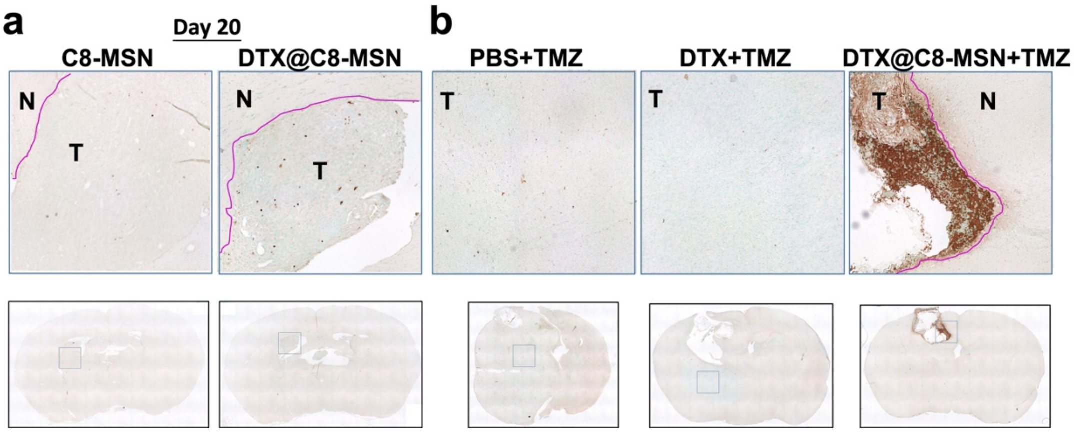


**Figure S5**. TUNEL staining for apoptotic cell detection in paraffin-embedded sections of transplanted GBM treated under various conditions. Apoptotic cells were stained as the brown signal. N: normal part; T: tumor part.
